# Regional Assessment of Temperature-Related Mortality in Finland

**DOI:** 10.3390/ijerph15030406

**Published:** 2018-02-27

**Authors:** Reija Ruuhela, Otto Hyvärinen, Kirsti Jylhä

**Affiliations:** Finnish Meteorological Institute, P.O. Box 503, FI-00101 Helsinki, Finland; otto.hyvarinen@fmi.fi (O.H.); kirsti.jylha@fmi.fi (K.J.)

**Keywords:** temperature-related mortality, distributed lag models, regional differences, meta-analysis

## Abstract

The aim of this study was to assess regional differences in temperature–mortality relationships across 21 hospital districts in Finland. The temperature dependence of the daily number of all-cause, all-aged deaths during 2000–2014 was studied in each hospital district by using daily mean temperatures, spatially averaged across each hospital district, to describe exposure to heat stress and cold stress. The relationships were modelled using distributed lag non-linear models (DLNM). In a simple model version, no delayed impacts of heat and cold on mortality were taken into account, whereas a more complex version included delayed impacts up to 25 days. A meta-analysis with selected climatic and sociodemographic covariates was conducted to study differences in the relationships between hospital districts. A pooled mortality-temperature relationship was produced to describe the average relationship in Finland. The simple DLNM model version without lag gave U-shaped dependencies of mortality on temperature almost without exception. The outputs of the model version with a 25-day lag were also U-shaped in most hospital districts. According to the meta-analysis, the differences in the temperature-mortality relationships between hospital districts were not statistically significant on the absolute temperature scale, meaning that the pooled mortality–temperature relationship can be applied to the whole country. However, on a relative temperature scale, heterogeneity was found, and the meta-regression suggested that morbidity index and population in the hospital districts might explain some of this heterogeneity. The pooled estimate for the relative risk (RR) of mortality at a daily mean temperature of 24 °C was 1.16 (95% CI 1.12–1.20) with reference at 14 °C, which is the minimum mortality temperature (MMT) of the pooled relationship. On the cold side, the RR at a daily mean temperature of −20 °C was 1.14 (95% CI 1.12–1.16). On a relative scale of daily mean temperature, the MMT was found at the 79th percentile.

## 1. Introduction

Mortality-temperature association is often schematically described as a U-shaped (or V-, J-shaped) curve with a trough at a so-called minimum mortality temperature (MMT) and increasing mortality towards both hot and cold tails of the temperature distribution. However, the exposure–response relationship is quite complex because of the non-linear and delayed impacts of thermal stress on human health. The shape of this relationship also varies regionally and in relation to sociodemographic factors [1,2,3,4].

The MMT is lower in cooler compared to warmer climatic zones [5,6], meaning that differences in MMT across climatic zones can be considered as an indicator of acclimatization of a population to their typical climatic environment. According to Guo et al. [6], on the basis of data from 12 countries in different climatic zones, the MMT is found at approximately the 75th percentile of the temperature distribution, varying between the 66th and 80th percentiles. However, Tobias et al. [7] detected a much wider range for the MMT among cities in Spain. Earlier studies indicated that in Finland the MMT is about 12–17 °C, while, in Mediterranean countries, the lowest mortality is found at 22–25 °C [5,8,9].

No systematic climate zone dependence has been found otherwise in the shape of the temperature-mortality relationship, thus in the steepness of the slopes around the minimum mortality temperature [5,6]. However, for example Curriero et al. [10] found a strong association of the temperature–mortality relationship with latitude in U.S. cities. People in colder climates are more sensitive to high temperatures than people in warm climates. Meta-analyses across European and U.S. cities [11,12,13] have also indicated heterogeneity in temperature–mortality relationships. The risk varies by community and country [6], and differences in vulnerability and sensitivity of the population to temperature extremes depend also on socioeconomic and non-climatic environmental factors. For instance, in a study comparing U.S. cities, Hondula et al. [14] concluded that places with a greater risk had a developed urban environment, higher percentage of children, elderly, and minority residents, and lower income and educational attainment; however, the key explanatory variables varied from one city to another.

The effects of hot temperatures on mortality appear immediately on the same day and usually last a few days, while the effects of cold appear typically after a couple of days and last about 10 days [6,15,16] or even weeks [4]. The impacts of extreme temperatures vary seasonally, and for instance the impacts of heat waves in early summer may be more severe than later in the season. Short-term acclimatization to seasonal variation takes place typically within a couple of weeks [17]. Furthermore, the heat and cold stress may lead to a displacement of mortality, called “harvesting”, with death taking place earlier than it would have happened otherwise [2].

The complexity of the mortality–temperature relationship creates challenges for modelling and assessing the overall impacts of hot and cold stress on mortality. In simple statistical models, mortality and temperature on the same day are compared. However, in recent years, more complex distributed lag non-linear models (DLNM) have been developed and applied in studies on the mortality–temperature relationship [2,18,19,20,21]. DLNM has been applied in several multi-country, (e.g., [6,22]), and multi-city studies including Helsinki, the capital of Finland [23], and at the country level, e.g., in Estonia, a neighbouring country of Finland [24].

**Figure 1:**
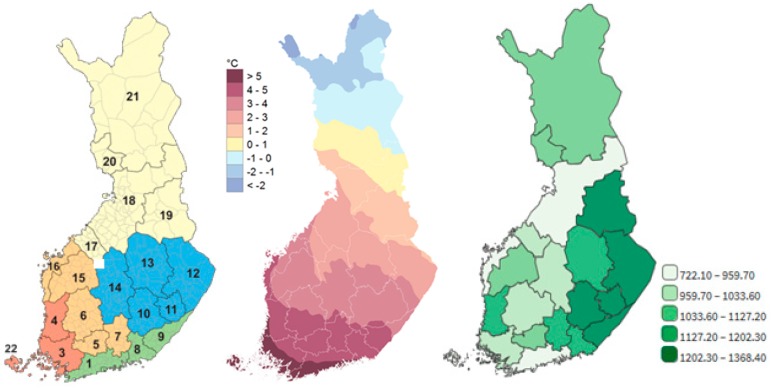
Hospital districts (**left**; 2011 regionalization), annual mean temperature in Finland in the climate normal period 1981–2010 (**middle**, [29]) and mortality (1/100,000) by hospital district in 2014 (**right**, [30]).

In vulnerability assessment of the elderly to climate change in Finland [25], one mortality-temperature relationship was applied to the whole country. However, there are substantial regional differences in mortality (Figure 1, right) and morbidity in Finland. According to a morbidity index of the National Institute of Health and Welfare in Finland, people in western and southern Finland are healthier than in eastern and northern Finland, and there are significant differences between municipalities in the prevalence of chronic disease [26]. The main motivation for modelling the mortality–temperature relationship regionally is to further improve the vulnerability assessment.

Thermal stress experienced by people depends not only on temperature but also on humidity, wind, and radiation balance. Thermal indices based on human energy balance describe the thermal environment more realistically than air temperature alone [27]. However, so far there is no consensus on the best thermal index for predicting temperature-related mortality. Based on a comparison of two indices, Physiologically Equivalent Temperature (PET) and outdoor air temperature, Ruuhela et al. [28] concluded that although PET appeared to better describe impacts of cold stress, the latter was applicable as well. Therefore, in this study, temperature is used as the indicator for heat and cold stress.

Our initial hypotheses were that: (1) there are regional differences in the mortality–temperature relationships between hospital districts in Finland; (2) these differences can be partly explained by climatic and sociodemographic factors. In this paper, we show that these hypotheses could not be fully confirmed. Furthermore, we study applicability of spline modelling methods in sparsely populated areas using models with and without delayed temperature impacts.

## 2. Materials and Methods 

### 2.1. Data

The regional administrative level of health services in Finland consists of 21 hospital districts (HD) (Figure 1, left). All-cause daily number of deaths and annual population in the HDs for the study period of 2000–2014 were obtained from Statistics Finland. Finland is a sparsely populated country with a somewhat higher population density in the southern part of the country. The population is highest, about 1.5 million, in the Helsinki-Uusimaa hospital district (HD1) (Supplementary Materials Table S1). In five hospital districts, the population is less than 0.1 million and, in the remaining hospital districts, it varies between 0.1 and 0.5 million. The share of elderly (75 years of age and older) in the hospital district population varies from 5% to 10%, and the highest values are found in eastern Finland HDs. The daily number of deaths in HD1 varied between 11 and 57, with a median of 30 deaths per day during our study period (Table S1). In the smallest hospital districts, the median of daily deaths was less than five, while in most of the hospital districts the median of daily deaths was about 10 with a maximum of around 20 deaths per day. In meta-regression analysis we also used hospital district-level morbidity indices from the National Institute for Health and Welfare (THL). In these indices, selected disease groups are weighted on the basis of their significance for mortality, disability, quality of life, and health-care costs in the population [26,31]. The morbidity index varied between 66 and 147, with the highest values in eastern Finland hospital districts.

Daily numbers of deaths were compared to daily mean temperatures that were calculated as a spatial average over each hospital district on the basis of a FMI (Finnish Meteorological Institute) daily gridded (10 km × 10 km) temperature data. The gridded data were produced by kriging interpolation, in which topography and water bodies are taken into account besides the measured temperature observations [32]. As the distance from the southernmost to the northernmost point in Finland is more than 1000 km, there are substantial temperature differences between southern and northern parts of the country (Figure 1, middle). In southwestern hospital districts, the spatially averaged daily temperature varied between –25 °C and +25 °C during our study period. More continental climatic conditions, with a wider temperature range, are found in eastern and northern hospital districts, e.g., in Northern Carelia (HD12 = Pohjois−Karjala) the range was from –35 °C to +27 °C. The spatial temperature variation on the daily level within each hospital district greatly depends on the prevailing weather conditions. The spatial temperature differences may be negligible, less than 0.5 °C, during cloudy weather, while the differences may be more than 20 °C in clear sky conditions during winter in Lapland, largely because of topographic variations in the region. 

### 2.2. Methods

In modelling the relationship between the number of daily deaths and daily mean temperatures in the hospital districts, we applied the distributed lag non-linear model (DLNM) and quasi-Poisson distribution for the number of deaths [19,20,33]. The general model definition is:

$$g({µ}_{t})=\alpha +s(x_{t};\beta )+\sum_{j=1}^{J}h_{j}(c_{ti};\gamma_{i})$$

where g is a log link function of the expectation µ_t_ ≡ E(Y_t_), with Y_t_ being the time series daily mortality counts in hospital district, α is an intercept, s(x_t_;β) is an exposure-response function to temperature (x_t_) defined by β and it is chosen as quadratic B-spline defined by internal knots. In addition, the cross-basis matrix of coefficients also describes lag effects of temperature, defined by knots for lag on a logarithmic scale. Here, we studied delayed temperature effects on mortality with a lag of up to 25 days. Confounding factors (c_ti_) in this study were day of the week and elapsed time from the beginning of the time series. Day of the week is modelled as a categorical variable and elapsed time as a natural cubic spline with 7 degrees of freedom (df) per year to control seasonal variation and long-term trend.

We applied different versions of DLNM, with varying lags and internal knots and with or without confounding factors. A simple version of the model, with no delayed temperature impacts (lag = 0), no confounding factors, and 4 internal knots for temperature distribution, was first applied for each hospital district (HD) separately. After this first-stage modelling, we studied potential regional differences in mortality–temperature relationship between the hospital districts with the aid of meta-regression analysis, following the method developed by Gasparrini et al. [34]. In order to reduce the effects of random variation in the relationships, especially in the less populated HDs, best linear unbiased predictions (BLUP) were applied. These BLUP estimates converge the HD-specific relationships towards a pooled, averaged exposure–response relationship. In the meta-analysis, Cochran Q test and I^2^ were used to study heterogeneity across the BLUP estimates of the relationships in hospital districts. Because temperature ranges deviate between the hospital districts, the meta-regression was done on both absolute and relative temperature scales.

We used selected characteristics of the hospital districts as covariates in meta-regression in order to assess their effect in explaining potential heterogeneity in temperature-mortality relationships between hospital districts. These covariates were climatological mean temperature, ranges of daily mean temperatures, morbidity indices, population, and share of elderly (75 years of age and older) in the hospital districts. LR test and Wald test were also applied to study the significance of these covariates to explain heterogeneity.

For sensitivity analysis of the modelled temperature-mortality relationship, we used three slightly different versions of the simple model: (i) three knots for temperature range; (ii) a model with the confounding effects of weekday and seasonal variation and long-term trend; (iii) a model with an exposure term of a moving average of daily mean temperature on the same and five previous days. The last analysis covers part the lagged effects of the heat and cold exposure.

For studying the impacts of temperature on mortality with long delay, we applied a more complex DLNM with lag up to 25 days. Here, the first modelling was performed using the lowest Akaike Information Criteria (AIC) value as a criterion for selecting the number of knots for the model in each hospital district. Thereby, the lag structure and optimal knots for the temperature distribution could be determined. The AIC-based number of knots varied from only one knot for both temperature and lag, to five knots for temperature and three knots for lag. On average, the best models had three knots for temperature and two knots for lag, and in the following step the modelling was done using these fixed numbers of knots for each hospital district.

The temperature–mortality relationships were concretized by reporting the relative risks of mortality (RR), with 95% confidence intervals (CI) at selected values (+24, +20, –15, –20, and –25 °C) of spatially averaged temperature (Tavg). The baseline for RR is the mortality at minimum mortality temperature. MMT and its 95% confidence intervals (CI) were determined with the help of a simulation method developed in Tobias et al. [7]. Furthermore, the lag distributions were drawn from the DLNM output. The R packages dlnm [20] and mvmeta [34] were used to conduct the studies.

## 3. Results

### 3.1. Model without Lag and Meta-Analysis

**Figure 2:**
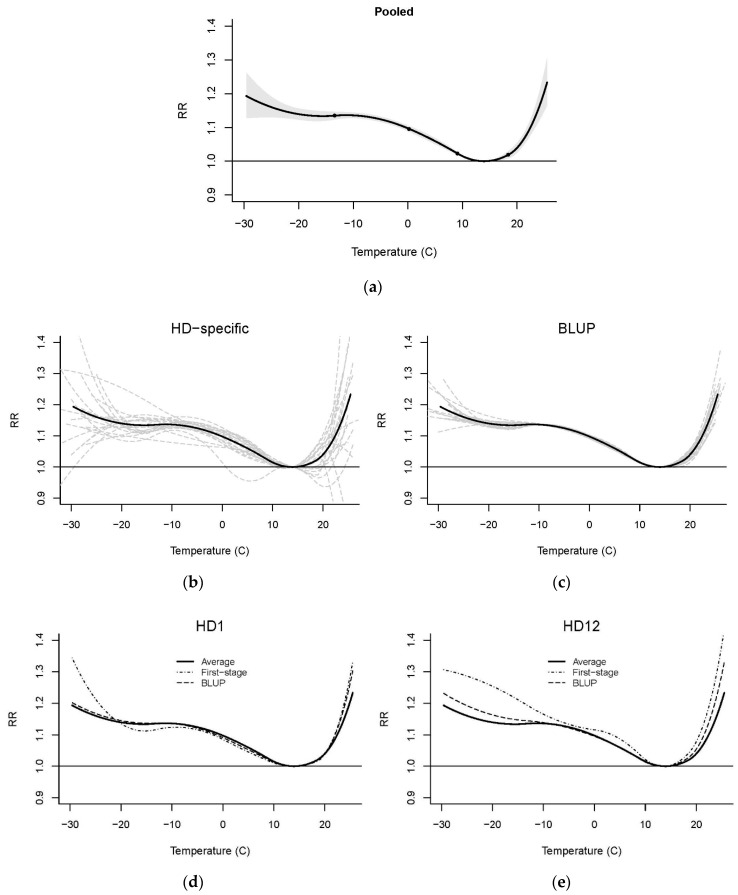
Temperature-mortality relationships based on the model without lag. (**a**) Pooled, average relationship (solid curve) with 95% confidence interval (shaded), dots indicating location of knots (up), relative risk (RR) reference at T = 14 °C; (**b**) hospital district (HD)-specific models (dash curves) with the pooled relationship (solid curve); (**c**) best linear unbiased predictions (BLUP) estimations for hospital districts. Examples on how BLUP estimation pulls the relationships towards the average in two hospital districts: (**d**) HD1, in southern Finland and (**e**) HD12, in eastern Finland.

The simple model version without lag produced U-shaped relationships in all hospital districts, except for the two least-populated ones, which have populations of less than 50,000. Another district, HD22, located in an archipelago in the Baltic Sea, was left out from the subsequent analysis. In Figure 2b, hospital district-specific mortality–temperature relationships, based on the first-stage modelling, are presented together with the pooled, average relationship. The HD-specific, first-stage modelling results show large differences and variation, especially on the cold temperature scale, but the BLUP estimation converges the relationships towards the average, and the differences between hospital districts almost disappear (Figure 2c). Examples of how the BLUP estimations differ from the first-stage models and from the pooled average in the hospital districts are presented in Figure 2d,e. In the Helsinki–Uusimaa hospital district (HD1), all the three relationships are fairly similar for typical temperatures, but in the cold extreme of the temperature distribution (below −20 °C), the first stage model outcome is remarkable higher than the BLUB estimate. In the less populated HD12 in eastern Finland, the BLUP estimate is drawn towards the average throughout the temperature scale more strongly than in HD1.

Exposure to high or low temperatures increased the mortality risk: the pooled estimate for the relative risk (RR) of mortality at a daily mean temperature of 24 °C was 1.16 (95% CI 1.12–1.20) when compared to mortality at 14 °C, which is the MMT of the pooled relationship. On the cold side, at a daily mean temperature of −20 °C, RR was 1.14 (95% CI 1.12–1.16). On the relative scale of daily mean temperature, the MMT was at the 79th percentile.

**Figure 3:**
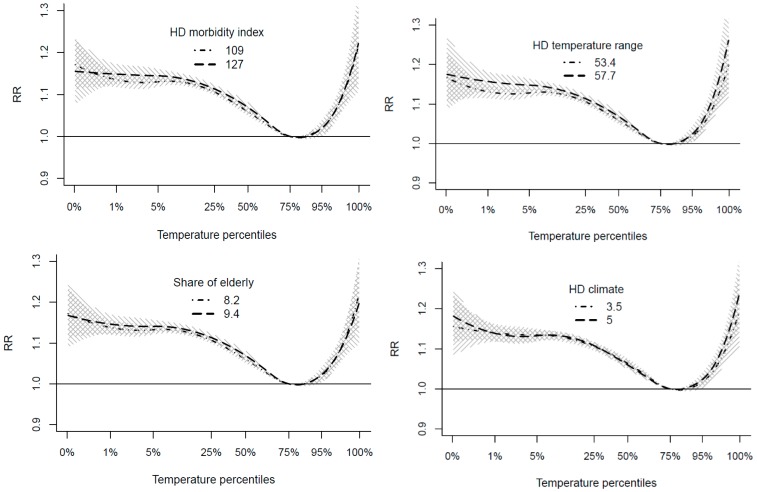
Meta-analysis of the temperature–mortality relationship on the relative temperature scale. Modelled relationships with the 25th and 75th percentiles of hospital district-level covariates: climatological mean temperature (°C), temperature range (°C), share of elderly (%), and morbidity index.

The pooled relationship had relatively narrow confidence intervals (Figure 2a), and the meta-analysis confirmed that there was no statistically significant heterogeneity in mortality–temperature relationships among the hospital districts when the analysis was made on an absolute temperature scale (Table 1). However, on the relative temperature scale, heterogeneity was found (Q test *p* = 0.029), and 21% of variation in the relationships would be explained by heterogeneity (I^2^ = 21.1%). According to the Wald test, morbidity index and population in the hospital districts explain heterogeneity on a statistically significant level, but the LR tests do not support these findings. The climatological mean temperature and temperature range do not explain a considerable amount of heterogeneity. Figure 3 demonstrates the small impacts of selected meta-regression covariates and the differences in mortality–temperature relationship at the 25th and 75th percentiles of the covariate. On the basis of this meta-analysis, we can conclude that, since there are no statistically significant differences in temperature–mortality relationships between hospital districts on the absolute temperature scale, the same relationship can be applied in all parts of the country. On the other hand, the meta-analysis on the relative temperature scale suggests that morbidity index and population in the hospital districts might explain some of the small regional heterogeneity of the temperature–mortality association. 

The sensitivity analysis of the modelled temperature–mortality relationships supports the finding above that there is no statistically significant heterogeneity in the temperature–mortality relationship across the hospital districts on the absolute temperature scale, while some heterogeneity is found on the relative temperature scale. Sensitivity analysis was performed using the simple model relationship with different parameterizations, and pooled outcomes of the results are presented in Supplementary Materials (Figure S1).

### 3.2. Model with 25-Day Lag

**Figure 4:**
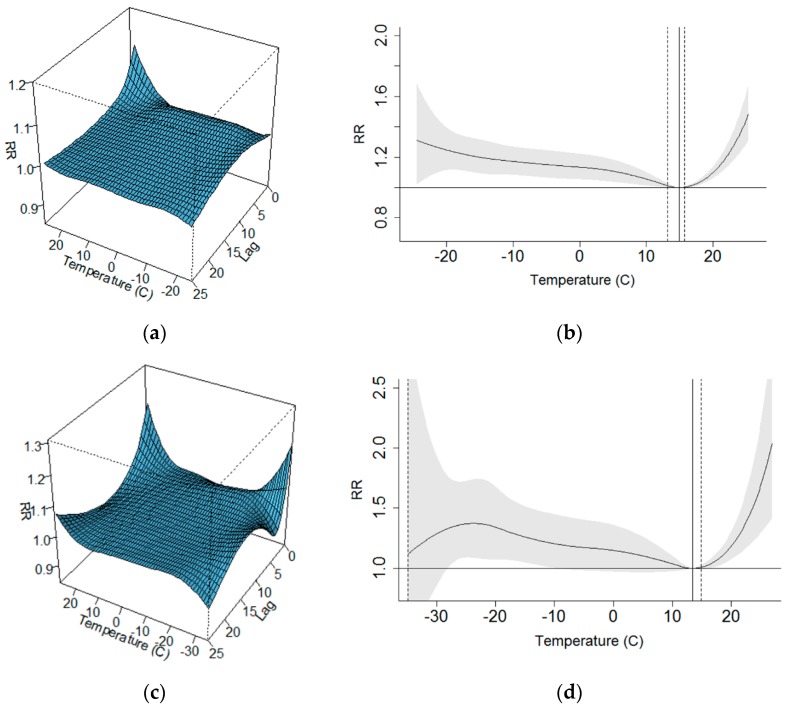
Outcomes of DLNM for hospital districts HD1 and HD12 with a model version with 25-day delayed temperature effects: (**a**,**c**) 3D visualizations of the relative risk of mortality as a function of temperature and lag; (**b**,**d**) overall relative risk (curves) with 95% CI (shaded). The point of minimum mortality temperature is indicated with a solid vertical line (95% CI, dotted vertical lines). HD1 is in southern and HD12 in eastern-central Finland. Modelling with fixed number of knots: 3 for temperature and 2 for lag.

The modelling with the more complex DLNM version with 25-day lag, day of the week, seasonal variation, and long-term trend as confounding factors, gave realistic U-shaped relationships in most of the hospital districts, even in those with a low population. A downside is that increased complexity in the DLNM version led to increased uncertainty in the model outcomes. In a few hospital districts, the modelled relationships appeared unrealistic, e.g., with continuously decreasing mortality with increasing temperature. Examples of 3D visualizations of the DLNM outputs and overall effects of temperature on the relative risk, aggregated from the 25-day delayed effects, are presented from two hospital districts in Figure 4. These hospital districts represent different climatic conditions. Helsinki–Uusimaa, HD1, is located in southern Finland, and its climate is affected by the Baltic Sea. During our study period, the daily mean temperature as a spatial average in the HD1 varied between −24.5 °C and +25.6 °C, with a median of 5.7 °C. Pohjois-Karjala, HD12, in eastern central Finland, has a more continental type of climate with a wider temperature variation range: the daily mean temperature varied between −34.1 °C and +26.9 °C, with a median of 3.2 °C. 

**Figure 5:**
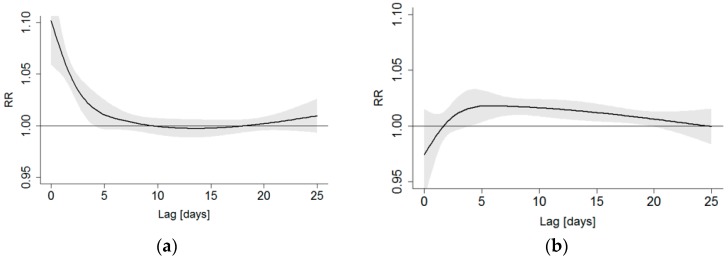
Examples of the effects of lag on relative mortality risk (RR) at two different temperatures, (**a**) Tavg = 24 °C, representing heat stress and (**b**) Tavg = −20 °C, cold stress. Examples of all-aged mortality in HD1, Helsinki–Uusimaa hospital district. DLNM with 3 knots for temperature and 2 for lag. Tavg: spatially averaged daily mean temperature.

Delayed impacts had common characteristics in most of DLNM outcomes: increase in the heat-related RR was apparent immediately on the same day and it disappeared within a few days. The cold-related RR appeared after a few days but lasted for several days or even weeks. Aggregating delayed impacts over 25 days increased the cold impact more than the heat impact. Examples of the structure of the lagged impacts of heat stress (at T = +24 °C) and cold stress (at T = −20 °C) on the all-aged mortality in HD1 are presented in Figure 5.

Examples of how inclusions of the delayed impacts may affect the estimated relative risks of all-aged mortality at various temperature levels are in Table 2 for two hospital districts. The outcomes of three model versions are presented: (i) the pooled model results without lag and without seasonal variation; (ii) hospital district-specific simple models without lag and without seasonal variation; (iii) a complex DLNM model with 25-day lag, seasonal variation, and a fixed number of knots for temperature and for lag. Our preliminary results suggest that including delayed impacts with long lag may double or triple the mortality risks, but since the meta-analysis of the complex model version was beyond this study, we cannot make firm conclusions from this model version.

## 4. Discussion

We conducted regional assessments of the temperature–mortality relationship in hospital districts in Finland by using different versions of distributed lag non-linear models (DLNM) and meta-analysis. Finland is a sparsely populated country, which generates challenges and large uncertainties in modelling. Earlier studies have focused on the capital city of Helsinki [11,23] or on larger areas such as southern Finland or northern Finland [5,8,9]. 

To our knowledge, this is the first attempt to model the temperature–mortality relationships at the hospital district level, i.e., at the regional administrative level of health services in Finland. The characteristics—e.g., area, population, public health, and climate—of hospital districts vary substantially, and our aim was to assess how heat- and cold-related health risks vary between hospital districts. 

Following the conclusions in Ruuhela et al. [28], we used the spatially averaged daily mean temperature in each hospital district as an indicator for thermal environment. Spatially averaged temperatures describe the thermal environment in a larger area better than single station-wise temperatures, since they include the topographic effects on the temperature variation in the area. We used 10 km × 10 km gridded climate data from the Finnish Meteorological Institute [30]. 

We found that modelling at the hospital district level gives realistic, U-shaped relationships even in sparsely populated areas, but with some limitations: the outcomes of the model version without lag were unrealistic in two hospital districts, possibly because of a small population, of less than 50,000, in those hospital districts. When complexity in modelling was increased by taking into account 25-day lag impacts of heat and cold, the uncertainties in the temperature-mortality relationships increased, and their shapes became unrealistic in a few more hospital districts in the northern and western part of the country. However, in regions where the modelling with a 25-day lag is possible, these models can provide a better understanding of the overall health impacts of heat and cold than the model without lag.

Studies that have found heterogeneity in temperature–mortality relationships were made in larger areas such as Europe or the USA [11,12,13]. Our study concentrated only on one country, and we could not confirm heterogeneity across Finland on the absolute temperature scale. According to the meta-analysis and BLUP estimates of temperature–mortality relationships in the hospital districts, the small differences in relationships across hospital districts were not statistically significant and cannot be explained by climatic or sociodemographic factors. Therefore, a pooled, average mortality–temperature relationship can be applied throughout the country, e.g., in climate change impact studies.

However, on the relative temperature scale, some heterogeneity was detected in the temperature‒mortality association between the hospital districts. Sociodemographic factors such as morbidity index and population might partly explain these differences. Yet, we cannot firmly conclude these outcomes because of the contradicting outcomes of the statistical tests.

The minimum mortality temperature (MMT) in Finland is clearly lower than among populations in warmer climates. Based on the pooled, average temperature–mortality relationship, the MMT in Finland is 14 °C. In a relative temperature scale, the MMT is found at the 79th percentile of the daily mean temperature distribution, thus somewhat higher than in studies from other countries, (e.g., [6]). We did not find regional differences in MMT, thus we cannot conclude that there would be regional differences in acclimatization in the Finnish population either. One explanation for this conclusion could be internal migration, which is substantial in Finland, mainly towards the south. However, in their study about internal migration and mortality, Saarela and Finnäs [35] found that the birth region is a very decisive mortality determinant. One limitation in our study was that we were not able to conduct research at the individual level and track the place of birth for each individual.

The relative risk (RR) of mortality in the hot tail of the temperature distribution appeared quite similar regardless of the DLNM version. In the cold tail of the temperature distribution, the variation and uncertainty in the modelled relationship was larger than in the hot tail, at least partly because of the long-lasting delayed impacts, as can be seen in Figure 5b. In this study, we focused on studying regional differences in the temperature–mortality relationship and finding the location of MMT, but modelling with various versions of DLNM model provided interesting outcomes for further studies and discussions on cold-related mortality. The simple model without lag or controlling seasonality produces classic U-shaped association between temperature and mortality. When seasonality was included into the model as a confounding factor, the cold tail of the association became flat, thus mortality risk did not appear to depend on temperature on the cold side. When delayed impacts were included into the model, the cold-related mortality risk was again elevated (Figure S1). When extending the lag up to 25 days, the cold impacts become more pronounced (Figure 4). 

These various model versions indicate that more research is needed on the winter mortality and impacts of cold. The study of Ebi and Mills [36] questioned the assumption that seasonality with higher mortality in winter than in summer would be attributable to temperature. Higher winter mortality may also be related to other factors that vary seasonally, such as influenza epidemics [37]. A climatologically interesting candidate to partly explain excess winter mortality, especially in high latitude countries, might be vitamin D concentration, which typically varies seasonally depending on solar radiation. In the meta-analysis of Rush et al. [38], vitamin D status was inversely associated with all-cause mortality. Similar conclusion was reported also in Finland [39]. 

According to the morbidity index of the National Institute of Health and Welfare, there are significant regional differences in public health in Finland [26]. We expected that these regional health differences would reflect into the temperature–mortality relationships as well, but our study did not fully support that. In the future, the regional differences in health risks due to heat and cold could be studied using, as additional explanatory variables, also other relevant socioeconomic and health indicators, such as the incidences of weather-sensitive chronic diseases. However, the small number of cases would limit the statistical power of such studies.

In areas of northern Finland with larger differences in elevation, the spatially aggregated temperature data may not describe well the exposure to heat or cold stress, because valleys are more populated than higher-altitude areas. One way forward in modelling the mortality–temperature relationship might be using population-weighted gridded temperature data.

The outcomes of this study can be used, e.g., in increasing the awareness of temperature-related health risks in high-latitude countries and, specifically, in discussions on the potential benefits of early warning systems in the health sector in Finland.

## 5. Conclusions

Modelling temperature dependence of mortality by DLNM without delayed impacts produced U-shaped relationships even in sparsely populated areas if the population is over 50,000. The DLNM with a 25-day lag also produced U-shaped relationships in most of the hospital districts and could thus provide a better understanding of the delayed health impacts of heat and cold than the model without delayed effects.

We did not find statistically significant differences in the temperature–mortality relationships between hospital districts on the absolute temperature scale, thus no differences in acclimatization between hospital district populations were found. Therefore, a pooled national average temperature–mortality relationship can be applied throughout the country in studies about future climate change impacts on temperature-related mortality in Finland. However, on the relative temperature scale, some heterogeneity was found, and the meta-regression suggested that morbidity index and population in the hospital districts might explain part of the heterogeneity.

## Figures and Tables

**Table 1:** Heterogeneity tests on absolute and relative temperature scales. The hospital district (HD)-level meta-analysis covariates are the following: long-term climatological mean temperature; range of daily mean temperatures, THL’s morbidity index, population, and share of elderly (75+ years). Statistically significant *p*-values with bold.

Covariate	Cochran Q Test			I^2^	Information Criteria		LR Test			Wald Test		
	Q	df	*p*	(%)	AIC	BIC	Stat	df		Stat	df	
***Absolute scale***												
Intercept-only	129.2	114	0.156	11.8	−304.8	−229.6						
***Relative scale***												
Intercept-only	144.4	114	**0.029**	21.1	−301.0	−225.7						
Climate-Tmean	134.8	108	**0.041**	19.9	−294.0	−202.0	5.0	6	0.544	5.6	6	0.465
Temperature range	134.1	108	**0.045**	19.5	−297.6	−205.6	8.6	6	0.196	11.0	6	0.089
Morbidity index	129.1	108	0.081	16.4	−298.5	−206.5	9.5	6	0.146	13.5	6	**0.036**
Population	127.5	108	0.097	15.3	−298.5	−206.5	9.5	6	0.146	14.9	6	**0.021**
Share of elderly	131.3	108	0.063	17.7	−295.8	−203.9	6.9	6	0.335	11.4	6	0.077

**Table 2:** Comparison of the relative mortality risk, RR (95% CI), in HD1 and HD12 at selected temperatures in three model versions: pooled model without lag and seasonal variation, a simple model without lag and seasonal variation, a DLNM with 25-day lag and seasonal variation.

	Tavg	RR (95% CI)	
**Pooled Estimate**	24	1.16 (1.12, 1.20)	
**-Without Lag**	20	1.04 (1.03, 1.05)	
	−15	1.13 (1.12, 1.15)	
	−20	1.14 (1.12, 1.16)	
	−25	1.16 (1.13, 1.19)	
		**HD1**	**HD12**
**Simple Model**	24	1.15 (1.10, 1.20)	1.25 (1.14, 1.38)
**-Without Lag**	20	1.06 (1.04, 1.08)	1.10 (1.05, 1.15)
	−15	1.14 (1.11, 1.16)	1.21 (1.17, 1.26)
	−20	1.14 (1.09, 1.18)	1.25 (1.20, 1.31)
	−25	1.13 (1.06, 1.20)	1.29 (1.21, 1.39)
**DLNM**	24	1.34 (1.22, 1.48)	1.55 (1.24, 1.94)
**-With 25-Day Lag**	20	1.10 (1.06, 1.13)	1.18 (1.07, 1.31)
	−15	1.20 (1.09, 1.31)	1.26 (1.05, 1.52)
	−20	1.25 (1.11, 1.39)	1.34 (1.07, 1.68)
	−25	1.32 (1.00, 1.74)	1.37 (1.09, 1.72)

## Supplementary Materials

The following are available online at http://www.mdpi.com/1660-4601/15/3/406/s1, Table S1: Characteristics of the hospital districts in 2000–2014: daily number of deaths, population, share of elderly, morbidity index, and spatially averaged daily temperature in the hospital districts, Figure S1: Pooled temperature–mortality relationships with different model parameters.
