# Supplementary material for: Regional Assessment of Temperature-Related Mortality in Finland
Source: Int J Environ Res Public Health. 2018 Feb 27;15(3):406. doi: 10.3390/ijerph15030406 (PMC5876951; doi:10.3390/ijerph15030406)
Supplement: Supplementary file 1 [file ijerph-15-00406-s001.pdf]

## Supplementary Materials:

# Regional assessment of temperature–related mortality in Finland

**Reija Ruuhela, Otto Hyvärinen and Kirsti Jylhä**

**Table S1.** Characteristics of the hospital districts in 2000–2014: daily number of deaths, population, share of elderly, morbidity index and spatially averaged daily temperature in the hospital districts.

**Figure S1.** Pooled temperature–mortality relationships with different model parameters.

**Table S1.** Characteristics of the hospital districts in 2000–2014: daily number of deaths, population, share of elderly (75 years and older), morbidity index (2001–2014) and spatially averaged daily temperature in the hospital districts

| | Daily deaths, all | | | Population, all | | | Share of elderly, 75+ years [%] | | | Morbidity index | | | Daily mean T [°C] | | |
|---|---|---|---|---|---|---|---|---|---|---|---|---|---|---|---|
| Hospital district | Median | Min | Max | Median | Min | Max | Median | Min | Max | Mean | Min | Max | Median | Min | Max |
| HD1, Helsinki-Uusimaa | 30 | 11 | 57 | 1471550 | 1375772 | 1599390 | 5.6 | 5.0 | 6.4 | 83.0 | 76.7 | 88.0 | 5.7 | –24.5 | 25.4 |
| HD3, Varsinais-Suomi | 12 | 2 | 27 | 461928 | 449316 | 449316 | 8.5 | 7.3 | 9.9 | 108.6 | 94.9 | 116.1 | 5.7 | –25.2 | 25.7 |
| HD4, Satakunta | 7 | 0 | 20 | 227590 | 223983 | 234228 | 9.4 | 7.7 | 10.4 | 108.2 | 103.4 | 112.3 | 5.3 | –26.6 | 25.5 |
| HD5, Kanta-Häme | 5 | 0 | 16 | 170703 | 165190 | 175481 | 8.9 | 7.7 | 9.7 | 102.4 | 100.2 | 105.0 | 5.1 | –27.3 | 25.7 |
| HD6, Pirkanmaa | 12 | 1 | 27 | 472015 | 443763 | 500265 | 7.9 | 6.8 | 8.7 | 101.8 | 98.2 | 104.6 | 4.7 | –28.9 | 25.6 |
| HD7, Päijät-Häme | 6 | 0 | 17 | 210934 | 208837 | 213542 | 8.4 | 6.8 | 9.7 | 116.2 | 107.6 | 123.1 | 4.9 | –28.0 | 25.5 |
| HD8, Kymenlaakso | 5 | 0 | 16 | 176675 | 172908 | 181119 | 9.4 | 7.8 | 10.4 | 119.1 | 111.8 | 125.7 | 5.2 | –28.1 | 26.2 |
| HD9, Etelä-Karjala | 4 | 0 | 13 | 134169 | 131764 | 136524 | 9.5 | 7.7 | 10.8 | 113.1 | 105.2 | 117.4 | 4.6 | –30.9 | 26.9 |
| HD10, Etelä-Savo | 3 | 0 | 13 | 108212 | 103873 | 113157 | 10.1 | 8.4 | 11.0 | 128.3 | 123.3 | 130.7 | 4.4 | –28.8 | 26.4 |
| HD11, Itä-Savo | 1 | 0 | 8 | 47083 | 44051 | 50110 | 10.9 | 9.1 | 11.4 | 126.3 | 122.6 | 130.0 | 4.2 | –30.7 | 26.2 |
| HD12, Pohjois-Karjala | 5 | 0 | 16 | 171326 | 168896 | 177235 | 9.0 | 7.3 | 9.9 | 139.2 | 129.5 | 147.4 | 3.2 | –34.9 | 26.9 |
| HD13, Pohjois-Savo | 7 | 0 | 18 | 249184 | 247943 | 255451 | 9.0 | 7.1 | 9.9 | 141.1 | 137.4 | 145.5 | 3.6 | –30.8 | 27.8 |
| HD14, Keski-Suomi | 7 | 0 | 18 | 270457 | 265131 | 275360 | 8.1 | 6.6 | 9.2 | 111.7 | 106.6 | 121.9 | 4.0 | –29.7 | 26.6 |
| HD15, Etelä-Pohjanmaa | 8 | 0 | 21 | 198832 | 198242 | 201999 | 9.6 | 8.0 | 10.3 | 115.4 | 111.2 | 118.2 | 4.2 | –28.8 | 24.7 |
| HD16, Vaasa | 4 | 0 | 14 | 162864 | 161112 | 169652 | 9.2 | 8.2 | 9.7 | 93.9 | 87.2 | 97.4 | 4.7 | –27.7 | 24.0 |
| HD17, Keski-Pohjanmaa | 2 | 0 | 9 | 74703 | 74180 | 75494 | 8.3 | 6.8 | 9.2 | 112.7 | 105.1 | 116.9 | 3.7 | –32.4 | 24.8 |
| HD18, Pohjois-Pohjanmaa | 8 | 1 | 25 | 388683 | 369835 | 408536 | 6.4 | 5.2 | 7.4 | 121.7 | 110.9 | 127.6 | 2.4 | –33.2 | 24.5 |
| HD19, Kainuu | 2 | 0 | 11 | 80477 | 76119 | 86940 | 9.2 | 7.1 | 9.8 | 134.2 | 131.4 | 135.9 | 2.2 | –32.2 | 25.9 |
| HD20, Länsi-Pohja | 2 | 0 | 9 | 66042 | 63603 | 69240 | 8.5 | 6.6 | 9.3 | 131.2 | 116.9 | 141.6 | 2.1 | –33.4 | 24.6 |
| HD21, Lappi | 3 | 0 | 13 | 118620 | 118145 | 125105 | 7.7 | 5.6 | 9.3 | 119.3 | 115.2 | 125.4 | 0.5 | –31.6 | 22.5 |
| HD22, Ahvenanmaa | 1 | 0 | 6 | 27038 | 25706 | 25706 | 8.3 | 8.3 | 9.7 | 75.6 | 65.7 | 83.1 | 6.2 | –22.3 | 24.1 |

**Figure S1. Pooled mortality-temperature relationships with different model parameters.**

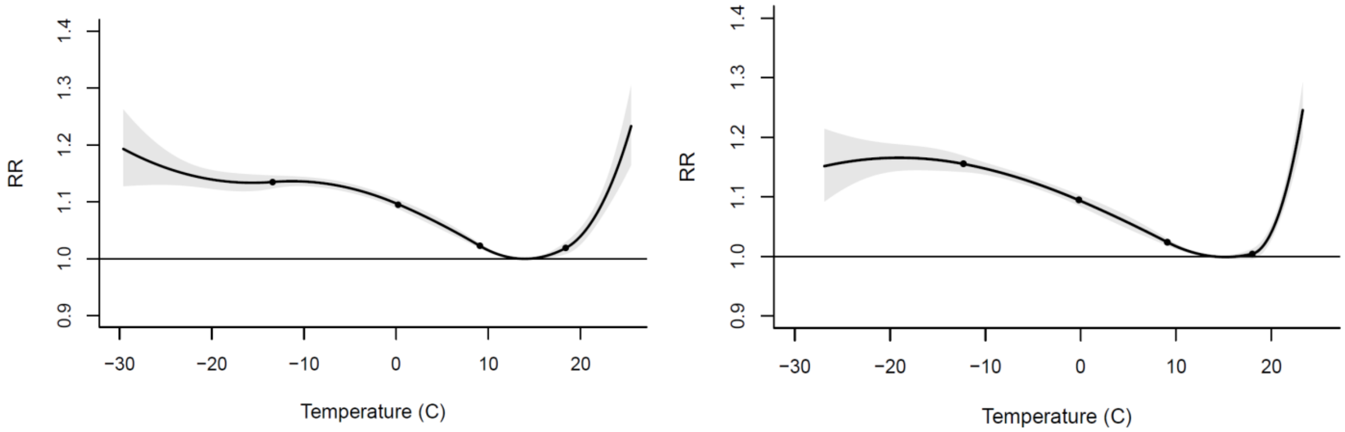


Exposure: Daily mean temperature (left), 5 day moving temperature average (right)

No confounding factors. Shaded areas represent 95% confidence intervals.

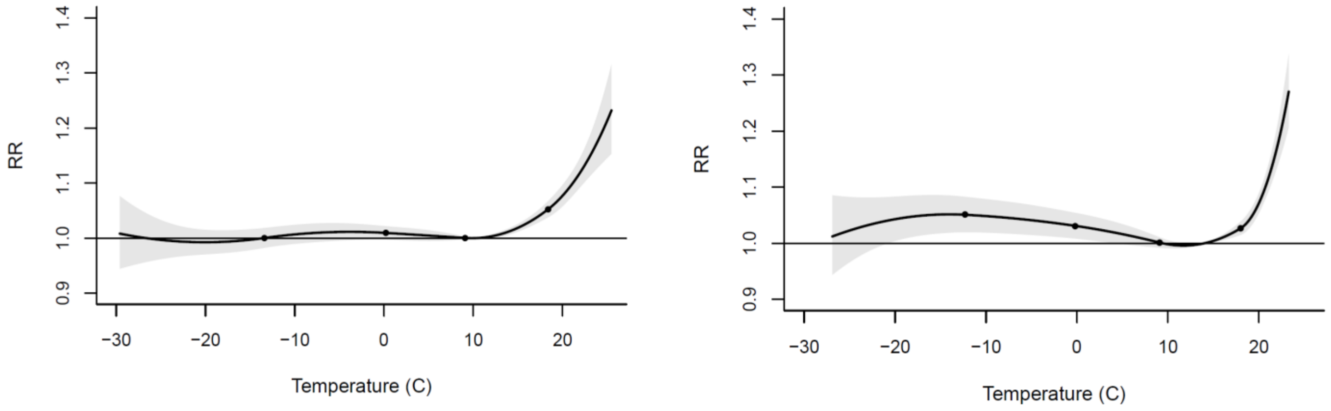


Confounding factors: day of the week and seasonal variation and long-term trend included;

Exposure: Daily mean temperature (left), 5 day moving temperature average (right)

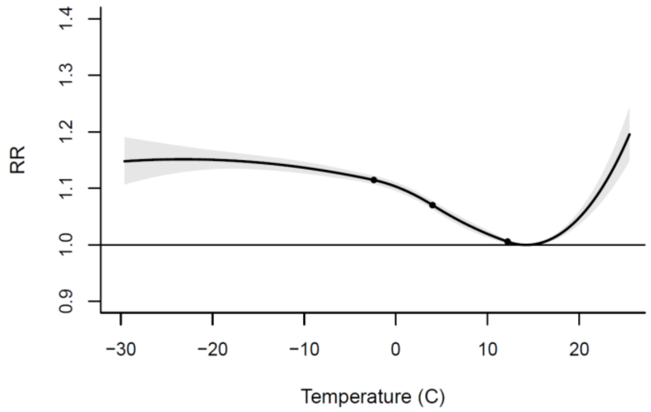


Exposure: Daily mean temperature, model with 3 internal knots for temperature. No confounding factors.
